# Salt-Tolerant Compatible Microbial Inoculants Modulate Physio-Biochemical Responses Enhance Plant Growth, Zn Biofortification and Yield of Wheat Grown in Saline-Sodic Soil

**DOI:** 10.3390/ijerph18189936

**Published:** 2021-09-21

**Authors:** Udai B. Singh, Deepti Malviya, Shailendra Singh, Prakash Singh, Abhijeet Ghatak, Muhammad Imran, Jai P. Rai, Rajiv K. Singh, Madhab C. Manna, Arun K. Sharma, Anil K. Saxena

**Affiliations:** 1 Plant-Microbe Interaction and Rhizosphere Biology Lab, ICAR-National Bureau of Agriculturally Important Microorganisms, Kushmaur, Maunath Bhanjan 275103, India; deeptimalviya77@gmail.com (D.M.); singh.shailendra512@gmail.com (S.S.); arun.dwr@gmail.com (A.K.S.); saxena461@yahoo.com (A.K.S.); 2 Department of Plant Breeding and Genetics, Veer Kunwar Singh College of Agriculture, Bihar Agricultural University, Dumraon, Buxar 802136, India; prakash201288@gmail.com; 3 Department of Plant Pathology, Bihar Agricultural University, Sabour, Bhagalpur 813210, India; ghatak11@gmail.com; 4 Department of Biosciences, Faculty of Science, Integral University, Lucknow 226026, India; imranm@iul.ac.in; 5 Department of Mycology and Plant Pathology, Institute of Agricultural Sciences, Banaras Hindu University, Varanasi 221005, India; drjaibhu@gmail.com; 6 ICAR-Indian Institute of Seed Sciences, Kushmaur, Maunath Bhanjan 275103, India; rajivsingh@iari.res.in; 7 Soil Biology Division, ICAR-Indian Institute of Soil Science, Bhopal 462038, India; madhabcm@gmail.com

**Keywords:** *Trichoderma harzianum*, *Bacillus amyloliquefaciens*, antioxidant, ZIP transporters, Zn bio-fortification, wheat, saline-sodic soil

## Abstract

A wide range of root-associated mutualistic microorganisms have been successfully applied and documented in the past for growth promotion, biofertilization, biofortification and biotic and abiotic stress amelioration in major crops. These microorganisms include nitrogen fixers, nutrient mobilizers, bio-remediators and bio-control agents. The present study aimed to demonstrate the impact of salt-tolerant compatible microbial inoculants on plant growth; Zn biofortification and yield of wheat (*Triticum aestivum* L.) crops grown in saline-sodic soil and insight of the mechanisms involved therein are being shared through this paper. Field experiments were conducted to evaluate the effects of *Trichoderma harzianum* UBSTH-501 and *Bacillus amyloliquefaciens* B-16 on wheat grown in saline-sodic soil at Research Farm, ICAR-Indian Institute of Seed Sciences, Kushmaur, India. The population of rhizosphere-associated microorganisms changed dramatically upon inoculation of the test microbes in the wheat rhizosphere. The co-inoculation induced a significant accumulation of proline and total soluble sugar in wheat at 30, 60, 90 and 120 days after sowing as compared to the uninoculated control. Upon quantitative estimation of organic solutes and antioxidant enzymes, these were found to have increased significantly in co-inoculated plants under salt-stressed conditions. The application of microbial inoculants enhanced the salt tolerance level significantly in wheat plants grown in saline-sodic soil. A significant increase in the uptake and translocation of potassium (K^+^) and calcium (Ca^2+^) was observed in wheat co-inoculated with the microbial inoculants, while a significant reduction in sodium (Na^+^) content was recorded in plants treated with both the bio-agents when compared with the respective uninoculated control plants. Results clearly indicated that significantly higher expression of *TaHKT-1* and *TaNHX1* in the roots enhances salt tolerance effectively by maintaining the Na^+^/K^+^ balance in the plant tissue. It was also observed that co-inoculation of the test inoculants increased the expression of ZIP transporters (2–3.5-folds) which ultimately led to increased biofortification of Zn in wheat grown in saline-sodic soil. Results suggested that co-inoculation of *T. harzianum* UBSTH-501 and *B. amyloliquefaciens* B-16 not only increased plant growth but also improved total grain yield along with a reduction in seedling mortality in the early stages of crop growth. In general, the present investigation demonstrated the feasibility of using salt-tolerant rhizosphere microbes for plant growth promotion and provides insights into plant-microbe interactions to ameliorate salt stress and increase Zn bio-fortification in wheat.

## 1. Introduction

Seven percent of the world’s total arable land (955 million ha) is affected by salt [1,2]. It has been estimated that about 7 million ha of land in India is salt-affected, including saline and alkali soils [3]. The Indo-Gangetic region alone has approximately 2.7 million ha of the area affected by salt [4] which is unfit for crop production due to high pH, low organic matter, and high concentrations of soluble salts such as Na_2_CO_3_ and NaHCO_3_, together with sufficient exchangeable sodium that causes poor physical soil characteristics. Salinity, being one of the major causes of loss in agricultural production in itself, has also led to considerable reduction in the area of arable land thus magnifying the degree of reduction in agricultural production to a greater extent [5]. Moreover, these soils are more prone to water-logging due to poor texture and permeability which affects crop establishment. Salt affected soils are inherently poor in microbial activities which, in turn, influences mineralization processes, uptake and translocation of essential nutrients in the plant system [6,7]. Excessive salt in the soil solution affects the absorption of water and nutrients and therefore, causes seedling mortality by pulling water from the root system (exo-osmosis). Further, the presence of excess sodium in saline and saline-sodic soil may induce deficiency of other cations like calcium, magnesium, potassium, zinc and iron. However, the high pH in sodic soils decreases the availability of many plant nutrients like N, P, Ca, Mg, Fe, Cu, Zn, etc. and thus adversely affects plant growth and development [8,9,10]. Among various micronutrients, availability of Zn is highly influenced under saline-sodic conditions. Zn is one of the key nutrients participating in major metabolic processes including synthesis of chlorophyll, indole-3-acetic acid (IAA) [11], carbohydrate, amino acids, protein and nucleic acid synthesis [12] and thereby influencing overall plant growth and development [13]. Several research indicated that Zn deficiency is recognized as a critical problem in plants, especially those grown in saline-sodic conditions with high pH. The Zn concentration decreased with elevated soil salinity/sodicity in many crops such as rice, wheat, and pepper plants [14,15,16]. The high salt concentration in the soil colloids reduces the uptake and translocation of Zn due to stronger competition by Na^+^ ions at the root surface [17]. Genc et al. [18] reported that Zn deficiency could be more important than sodium (Na^+^) and chloride (Cl^−^) toxicity causes poor plant growth and their establishment. The increased availability of Zn reduces the accumulation of Na^+^ ions in the plants system and thereby protects plants from salt injury and reactive oxygen species (ROS) through improved antioxidants system [19,20]. 

Wheat (*Triticum aestivum* L.) is the second most important staple food crop grown throughout the world after rice. Wheat forms a critical ‘stuff of life’ being the food for 2.5 billion people across 89 countries of the world and ranks first in terms of source of calories and second in terms of source of proteins in low- and middle-income countries like India, Nepal, Pakistan, Bangladesh, etc. [21,22,23]. However, wheat is exceptionally sensitive to salinity and sodicity in its early seedling stages [24,25] and high losses have been observed in yield because of higher rates of seedling mortality and poor crop establishment. Furthermore, modern high yielding varieties of wheat are considerably more sensitive to higher salt concentrations. The use of salt-tolerant cultivars is one of the sensible approaches to meet this problem and some cultivars with moderate salt tolerance have also been developed for this purpose. However, none of the cultivars having a high degree of salt-tolerance are commercially available so far. Therefore, we are still in need of donor parents with quantitative trait loci (QTLs) or gene(s) of salt tolerance to prevent losses caused by these stresses to our wheat production [26,27,28]. To date, the process and practice of desalinization have been largely dependent on the integration of pyrite, gypsum and modified agronomic practices. Adaptation of pyrite and gypsum requires considerable investment to re-claim these soils to obtain reasonable yields and this investment seems beyond the capacity of the resource-poor farmers living in the salt-affected areas. Increasing and sustaining crop production in these areas will require a well-proven system that integrates salt-tolerant varieties with effective and affordable crop and nutrient management practices [27,29,30].

In the recent past, attention has been given to identify and utilize the consortia of compatible salt-tolerant rhizospheric microorganisms (STRM) that can mediate induced systemic tolerance (IST) to sustain and improve plant growth under such stressful conditions [31,32,33]. STRM can improve plant growth through one or more mechanisms, either directly through providing essential nutrients, production of phytohormones and maintaining equilibrium between cations and anions, or indirectly through IST, which enables plants to tolerate or attenuate deleterious effects of higher salt concentrations [29,30]. Experimental evidence suggested that STRM promotes plant growth through regulation of osmotic balance, altering root size and morphology, enhancing nutrient uptake and translocation favourably [28,31]. In addition, certain plant growth-promoting rhizobacteria (PGPR) ameliorate ion-induced damage and improve plant growth through *HKT1* (high-affinity K^+^ transporter) gene expression in several crop plants under high salt conditions [29,34,35,36,37,38]. Few reports indicate that PGPR triggers localized and systemic cellular mechanisms/cascades in plants to protect them from high salt [38]. However, the regulatory mechanisms remain unexplained [34,39]. Further, it was also observed that limited reports are available on the microbe-mediated biofortification of Zn in wheat grown under salt-stressed condition. Among them, most of the studies were conducted under controlled laboratory or glasshouse conditions. The need of the hour is to evaluate the prospective salt-tolerant microbial inoculants under field conditions which will give a real picture of their potential through research. Therefore, understanding the microbe-mediated mechanisms of salt tolerance and the use of microbial inoculants is essential for solving the problem in enhancing crop productivity and Zn biofortification under high salt conditions. Keeping in view above said problems, the objective of the present investigation was determined to evaluate the salt-tolerant *Trichoderma harzianum* UBSTH-501 and *Bacillus amyloliquefaciens* B-16 with an attempt to decipher the mechanisms of induced systemic tolerance and their impact on plant growth, Zn-biofortification and yield of wheat crop grown in saline-sodic soil of warm humid Gangetic plains of India. The present investigations revealed the significance of microbial inoculants in improving the nutritional status of wheat crops even grown under salt-stressed conditions. These strains could be used as bio-inoculants for the biofortification of wheat to combat hidden hunger in developing countries. Biofortification of wheat crops by utilizing these potential microbial strains can be done to reduce malnutrition in the world. The current research works toward a comprehensive assessment of these strains for sustainable production of quality wheat across the globe.

## 2. Materials and Methods

### 2.1. Media, Chemical Reagents and Planting Materials

Dehydrated culture media and talc powder were procured from HiMedia, India, while analytical grade chemical reagents and standards were purchased from E. Merck, India.

Wheat seeds (*cv.* HUW 234) were obtained from ICAR-Indian Institute of Seed Science (ICAR-IISS), Kushmaur, Mau, India and evaluated under nethouse and field conditions.

### 2.2. Salt-Tolerant Microbial Strains

*Trichoderma harzianum* UBSTH-501 and *Bacillus amyloliquefaciens* B-16 were obtained from Plant-Microbe Interaction and Rhizosphere Biology Lab, ICAR-National Bureau of Agriculturally Important Microorganisms (ICAR-NBAIM), Kushmaur, Maunath Bhanjan, Uttar Pradesh, India. The fungal and bacterial strains were maintained on potato dextrose agar (PDA) and nutrient agar (NA) medium, respectively, at 27 °C by sub-culturing at 10 days intervals. The bacterium is being maintained in glycerol stock (−80 °C) and the fungal strain in mineral oil for long-term storage.

### 2.3. In Vitro Screening for Salt Tolerance and Plant Growth-Promoting Traits

The salt tolerance capability of *T. harzianum* UBSTH-501 and *B. amyloliquefaciens* B-16 was screened on PDA and NA media, respectively, supplemented with different concentrations of NaCl (0.5–10%) as per the methods described by Singh et al. [25]. The promising bioagents were further screened for their plant growth-promoting (PGP) traits, namely the production of IAA [40], siderophore [41], ammonia [42], and phosphate-solubilisation [43] using standard methods. The process of colorimetric estimation of protease was carried out as per the methods described by Boller and Mauch [44]. The production of HCN, H_2_O_2_, urease, catalase and starch hydrolysis test was performed as per the methods described in Bergey’s Manual of Systematic Bacteriology [45].

### 2.4. Mass Multiplication and Development of Bioformulations

Talc-based bioformulations of *T. harzianum* UBSTH-501 and *B. amyloliquefaciens* B-16 were prepared as per the methods described by Singh et al. [46]. The vermicompost-based formulation of *T. harzianum* UBSTH-501 was prepared as per the methods described by Singh et al. [47] with slight modifications. Briefly, *T. harzianum* UBSTH-501 was grown on sorghum grain and incubated for 20 days (until complete grains were covered with mycelium and spore mass of the test fungus). In the meantime, vermicompost was collected from the production site, air-dried, sieved (2 mm pore size) and moistened by sprinkling water (60%). Thereafter, the compost was treated with carbofuran (10g kg^−1^), covered with a black polythene sheet and incubated for 72 h under sunlight. Afterwards, such treated compost heap was opened and turned to release the remaining amount of carbofuran into the air. The next day, compost was treated with a grain-based formulation of *T. harzianum* UBSTH-501 (20 g kg^−1^), covered with autoclaved, moistened gunny bags and incubated for 10 days. On the 11th day, such bio-fortified compost was turned and mixed properly, again covered with moistened gunny bags and incubated for the next 10 days at ambient temperature (25 ± 2 °C) under shade. After 20 days, bio-fortified compost became colonized by *T. harzianum* and was ready for its field application. The colony-forming unit (CFU) of talc-based bioformulation of *B. amyloliquefaciens* B-16, *T. harzianum* UBSTH-501 and vermicompost-based formulation of *T. harzianum* UBSTH-501 were 1.85 × 10^8^, 2.10 × 10^6^ and 0.66 × 10^6^ g^−1^, respectively.

### 2.5. Evaluation of Microbial Inoculants

#### Effect of Seed Bio-Priming on Seed Germination and Vigour Indices under Nethouse Conditions

Effect of microbial inoculants on seed germination (%), vigour index I and vigour index II were assayed as per the methods suggested by International Seed Testing Association [48] with slight modifications [49]. In brief, seeds were surface-sterilized using sodium hypochlorite (NaOCl) according to Singh et al. [38]. The surface sterilized wheat seeds (*cv.* HUW 234) were bio-primed with the talc-based formulation of *B. amyloliquefaciens* B-16 (10g kg^−1^ seeds) and *T. harzianum* UBSTH-501 (10g kg^−1^ seeds) and sown in pots (25 × 25 cm) containing potting mixture (soil and vermi-compost in 2:1 ratio). Each pot contained 5 kg of sterilized potting mixture and moisture was maintained by sprinkling sterilized water at 5 days intervals. The growing conditions were average temperature 22–25 °C with relative humidity of 70–75% and photoperiod being 11/13 h. To assess vigour, the length and dry weight of the root and shoot of an individual seedling was measured 30 days after sowing (DAS) under nethouse conditions. The vigour index I and vigour index II were calculated using the formulae as described by Abdul-Baki and Anderson [50] and Kharbet al. [51], respectively with slight modification [46]. To ensure the consistency of data through statistical validity, the entire experiment was repeated three times in ten replicates of 100 seeds each and the average was taken for statistical analysis.

### 2.6. Evaluation of Microbial Inoculants under Field Conditions

#### 2.6.1. Experimental Set-Up

The experiments consisted of two different treatments: (i) plants inoculated with bioagents *T. harzianum* UBSTH-501 and *B. amyloliquefaciens* B-16; (ii) control. Experiments were repeated twice, and each treatment consisted of five replications under field conditions in a Randomized Block Design.

The rice-wheat cropping system is the predominant crop rotation in the experimental field. The coordinates of the two fields were 25°53′59.18″ N 83°29′17.29″ E and 25°53′58.26″ N 83°29′15.61″ E, respectively, with an elevation of 72 m above mean sea level. The experiments were repeated twice with three replications from November 2014 to April 2015 in two different neighbouring plots and pool analysis was done. The size of the individual plot was 15 × 10 m with a border space of 1 m. The chemical fertilizers were applied to supplement nitrogen, phosphorus and potassium in a proportion of 120:60:40 (N:P:K) kg ha^−1^. Along with NPK, 20 Kg of zinc sulphate was also applied as basal dressing during soil preparation. The physico-chemical properties of the initial soil are presented in Supplementary Table S1. Minimum tillage (reduced tillage) practice was adopted in which only two ploughings—one with a cultivator and the other with a rotavator—were done. For moisture management, first irrigation was given with the help of a sprinkler, whereas following irrigations were done by flooding to saturate the soil at 20-day intervals. The growing conditions included an average temperature of 22–25 °C and relative humidity of 70–75% with a photoperiod of 11/13 h.

Seeds were bio-primed with the talc-based formulation of *B. amyloliquefaciens* B-16 at 10 g kg^−1^ of seed, covered with a black plastic sheet and incubated for 24 h at ambient temperature (27 ± 2 °C). However, *T. harzianum* UBSTH-501 bio-fortified vermicompost was applied as a basal dressing (4 tonnes ha^−1^) 24 h before sowing. The bio-primed seeds were sown in the field with the help of a tractor-operated seed drill in the evening hours with a spacing of 10 × 15 cm in plots of dimensions 15 × 10 m. Field amended with untreated compost was taken as the control for *T. harzianum* UBSTH-501 and seeds treated only with plain talc were taken as the control for *B. amyloliquefaciens* B-16. The control plots thus had untreated compost in the soil and talc-treated seeds were sown into this plot to nullify the effect of vermicompost and talc powder, if any.

#### 2.6.2. Estimation of Log CFU Count

The soil samples from the wheat rhizosphere were collected at different time intervals (30, 60, 90 and 120 days after sowing). To collect the rhizosphere soil, twenty-five plants were uprooted and shaken gently. The soil adhered to the roots system was collected (10g) in a separate plastic bag. Soil samples, thus collected, were brought to the laboratory, air-dried in shade to remove the excess moisture up to the field capacity. The samples were sieved (2mm pore size) to remove the debris and fine soil particles were stored in a cold room at 8 °C. The cultivable fungal population was assayed by plating serial decimal dilution on corn meal agar, glucose agar, soil extract agar, Czapek-Dox agar and PDA; whereas cultivable rhizospheric bacterial population was enumerated on nutrient agar, bacillus agar, yeast glucose agar, King’s Medium B and soil extract agar. The inoculated plates were incubated at 27 ± 2 °C. However, the actinomycetes population was counted on starch casein agar, actinomycetes isolation agar, ISP-2, ISP-3, and ISP-4 with an amendment of calcium carbonate. For enumeration of actinomycetes, the inoculated plates were incubated at 28 °C. The log CFU was calculated on a dry weight basis (soil moisture of 60%).

#### 2.6.3. Estimation of IAA in Rhizosphere Soil

Indole-3-Acetic Acid (IAA) in the rhizosphere soil of wheat was estimated as per the methods described by Thimmaiah [52] at 30, 60, 90, and 120 DAS. Briefly, 1.0g rhizospheric soil was collected, dried under vacuum and suspended in methanol:water (1:1, *v*/*v*, 5.0 mL) thrice. The supernatants were pooled, and the solvent was evaporated. The dried extracts were re-dissolved in methanol and were subjected to filtration (cellulose nitrate filter, 0.2 µm) prior to analysis. IAA quantification in the rhizospheric soil extracts was done by the colorimetric method using UV Vis Spectrophotometer (Shimadzu Corporation, Japan).

#### 2.6.4. Effect of Bioagents on Membrane Thermostability, Chlorophyll Content and Accumulation of Biomolecules and Organic Solutes

To explore whether microbial inoculants elicit changes in the accumulation of total chlorophyll, total soluble sugar, total protein, starch content, and proline content in wheat grown in saline-sodic soil, time-course experiments were conducted in two contrast treatments, i.e., plants treated with microbial inoculants and untreated control. The changes in the accumulation of biomolecules were measured spectrophotometrically, as per the methods described by Sadasivam and Manickam [53], whereas membrane thermostability (%) was determined in the first fully expanded leaves as per methods described by Fokaret al. [54] at 30, 60, 90 and 120 DAS.

#### 2.6.5. Effect of Bioagents on Lipid Peroxidation and Antioxidant Enzymes Activity

The amount of lipid peroxidation (malondialdehyde, MDA) was measured following the methods described by Heath and Packer [55]. The changes in the accumulation and activity of catalase and superoxide dismutase in plant leaves were measured following the methods described by Thimmaiah [52], whereas the activity of peroxidase was estimated following the protocols described by Sadasivam and Manickam [53] at 30, 60, 90 and 120 DAS. For estimation of SOD, ground 1 g of fresh clean plant tissue in 10 mL ice-cold 50mM potassium phosphate buffer (pH 7.8) in a pre-chilled pestle and mortar. Centrifuged the homogenate at 10,000 rpm for 10 min at 4 °C in a refrigerated centrifuge. The supernatant is used as an enzyme source within 12 h of extraction. Mixed a 3mL reaction cocktail containing (50mM potassium phosphate buffer, pH 7.8, 13mM methionine, 2µM riboflavin, o.1 mM EDTA, 75 µM NBT and 50µL of crude enzyme extract, in duplicate. Make the volume equal by adding double distilled water. Further, set a blank without enzyme and NBT was taken to calibrate the spectrophotometer, while set another control having NBT but no enzyme as reference control. Finally, read the absorbance at 560 nm immediately. The enzyme activity is expressed as units/mg of protein. While quantitative estimation of peroxidase was done according to Sadasivam and Manickam [53] at 30, 60, 90 and 120 DAS. Briefly, 1 g of fresh plant tissue was ground in 3 mL of 0.1 M phosphate buffer (pH 7) by using a pre-cooled mortar and pestle. Centrifuged the homogenate at 18,000 (5 °C) for 15 min. The supernatant was used as an enzyme source. Further, pipetted out 3 mL buffer solution, 0.05 mL guaiacol solution, 0.1 mL enzyme extract and 0.03 mL hydrogen peroxide solution in a cuvette. It was mixed well, and observance was recorded using spectrophotometer at 436 nm. In a similar way, catalase was assayed following the steps suggested by Thimmaiah [52].

#### 2.6.6. Gene Expression Analyses

To see the effect of microbial inoculation on expression profile Zn transporter (*TaZIP*) genes in wheat grown in saline-sodic soil, total RNA was extracted from root, stem, leaf and panicles using a Total RNA Isolation Kit (Agilent, New Delhi, USA) following the manufacturer’s protocols at 90 DAS. cDNA was synthesized using iScript™ cDNA Synthesis Kit (Bio-Rad Laboratories, Haryana, India) according to the manufacturer’s protocols. The cDNA was quantify before qPCR by using NanoDrop™ 2000/2000c Spectrophotometer (Thermo Scientific, Waltham, MA, USA). Gene specific qPCR primers were designed (Table 1) and validated *in silico* (https://www.ncbi.nlm.nih.gov/tools/primer-blast/, accessed on 11 August 2021) prior to qPCR experiments. *Actin* and *SuccDH* genes were taken as internal control (reference) to normalize the expressions of the genes studied. The data of real-time qPCR were analysed using the 2^−ΔΔC^T method [56]. Expression analyses was carried out using MJ MiniOpticon Real-Time PCR System (Bio-Rad, Hercules, CA, USA) according to Singh et al. (2021). However, expression profile of High-Affinity K^+^ Transporter *(TaHKT*-1) and Sodium/Hydrogen exchanger 1 (*TaNHX*-1) genes was analysed in wheat grown in saline-sodic soil at 30, 60, 90, and 120 DAS.

#### 2.6.7. Estimation of Zn Bio-Accumulation in Wheat

The total Zn in the root and shoot was estimated using Atomic Absorption Spectrometer (Thermo Fisher Scientific, Waltham, MA, USA) following the standard protocols at 90 DAS [57]. However, Zn content in grains was estimated after harvest. Further, Bioaccumulation factor (the ratio of Zn concentration in plant biomass to Zn in soil), Translocation Factor (translocation of Zn from root to shoot), and Transformation Factor (translocation of Zn from shoot to grain) were calculated using the formulae given below:

Ma et al. [58]

$$\mathrm{Bioaccumulation}\;\mathrm{factor}\;(\mathrm{BAF})=\frac{\mathrm{Zn}\;\mathrm{concentration}\;\mathrm{in}\;\mathrm{plant}\;\mathrm{biomass}\left(\mathrm{above}\;\mathrm{ground}\right)}{\mathrm{Zn}\;\mathrm{concentration}\;\mathrm{in}\;\mathrm{soil}}$$

Baker and Brooks [59]

$$\mathrm{Translocation}\;\mathrm{factor}\;(\mathrm{TF})=\frac{\mathrm{Zn}\;\mathrm{concentration}\;\mathrm{in}\;\mathrm{plant}\;\mathrm{shoot}}{\mathrm{Zn}\;\mathrm{concentration}\;\mathrm{in}\;\mathrm{plant}\;\mathrm{root}}$$

Present study

$$\mathrm{Transformation}\;\mathrm{factor}\;(\mathrm{TrF})=\frac{\mathrm{Zn}\;\mathrm{concentration}\;\mathrm{in}\;\mathrm{grain}}{\mathrm{Zn}\;\mathrm{concentration}\;\mathrm{in}\;\mathrm{shoot}}$$

#### 2.6.8. Plant Growth Promotion and Yield

The effects of microbial inoculants on plant growth, yield and yield attributing characteristics were measured in the wheat crop grown in saline-sodic soil. Ten plants from each treatment were sampled to measure the average plant height, number of effective tillers per plant and plant biomass (dry weight) at 30, 60, 90 and 120 DAS. The crop growth rate (g cm^−2^ day^−1^) was measured and calculated as per the methods described by Watson [60]. Data on yield and yield attributes were collected after harvest. Ten plants from each treatment were sampled to calculate the spike length (cm), spike weight (g), spikelet spike^−1^, number of seeds spike^−1^ and seed weight (g) spike^−1^. To record grain yield (qha^−1^), straw yield (qha^−1^) and total biological yield (qha^−1^), sampling was done from five random places in each treatment using a quartet of 1 m^2^ and finally, yield was calculated on ha^−1^ basis. The random sampling was done to calculate the weight (g) of 1000 seeds (denoted as test weight) for each treatment according to ISTA guidelines [48].

#### 2.6.9. Effect of Bioagents on Na^+^, K^+^ and Ca^++^ Uptake in Plant

To see the effect of microbial inoculation on uptake of Na^+^, K^+^ and Ca^++^, 10 plants were sampled randomly from each treatment at 30, 60, 90 and 120 DAS, dried at 60 °C and digested using standard protocols. The content of Na^+^, K^+^ and Ca^++^ was estimated as per the methods described by Singh et al. [25].

### 2.7. Statistical Analyses

Nethouse and field experiments were laid out in randomized block design (RBD) in three replications. The analysis of the data collected from different experiments was done using the statistical package, StatisticalAnalysis System version 9.2 (SAS 9.2, North Carolina State University, Raleigh, NC, USA).

## 3. Results

### 3.1. In Vitro Screening for Salt Tolerance and Plant Growth-Promoting Traits

The selected strains were screened for their salt tolerance ability. The results showed that *B. amyloliquefaciens* B-16 grew in a medium containing 5%NaCl, whereas *T. harzianum* UBSTH-501 grew at a salt concentration of 4.5% only (Supplementary Table S2). Further, *B. amyloliquefaciens* B-16 was found to be positive for all the traits tested and *T. harzianum* UBSTH-501 was positive for HCN, IAA, siderophore, starch hydrolysis, protease production and P, K and Zn solubilization under in vitro assay (Supplementary Table S2).

### 3.2. Effect of Seed Bio-Priming on Seed Germination and Vigour Indices

The effect of selected bioinoculants on seed germination (%), vigour index I and II were studied under nethouse conditions at 30 DAS. They were found to increase seed germination (%) and vigour indices significantly as compared to the untreated control. Seeds treated with *T. harzianum* UBSTH-501 showed maximum germination (87.23%) as well as vigour indices I and II (3413.22 and 1.95, respectively) followed by *B. amyloliquefaciens* B-16 after 30 DAS. However, minimum seed germination (80.92%) and vigour indices (vigour index I: 3112.25 and vigour index II: 1.75) were recorded in the untreated control (Table 2).

### 3.3. Estimation of Log CFU Count

The effect of microbial inoculation on microbial population in the rhizosphere was studied at different growth stages of wheat crop (30, 60, 90 and 120 DAS) grown in saline-sodic soil. A continuous increase in the log CFU count was recorded up to 90 DAS and the maximum counts of bacterial, fungal and actinomycetes were recorded at the dough stage (90 DAS) in rhizosphere of the plants co-inoculated with *T. harzianum* UBSTH-501 and *B. amyloliquefaciens* B-16 (Table 3). After 90 DAS, the microbial count decreased at a faster rate. The minimum CFU count, however, was recorded at 30 DAS. A more or less similar trend was recorded in the rhizosphere of control plants. Results also indicated that CFU count of fungi and actinomycetes did not differ significantly from those in control at 30 and 60 DAS (Table 3).

### 3.4. Estimation of IAA

**Figure 1:**
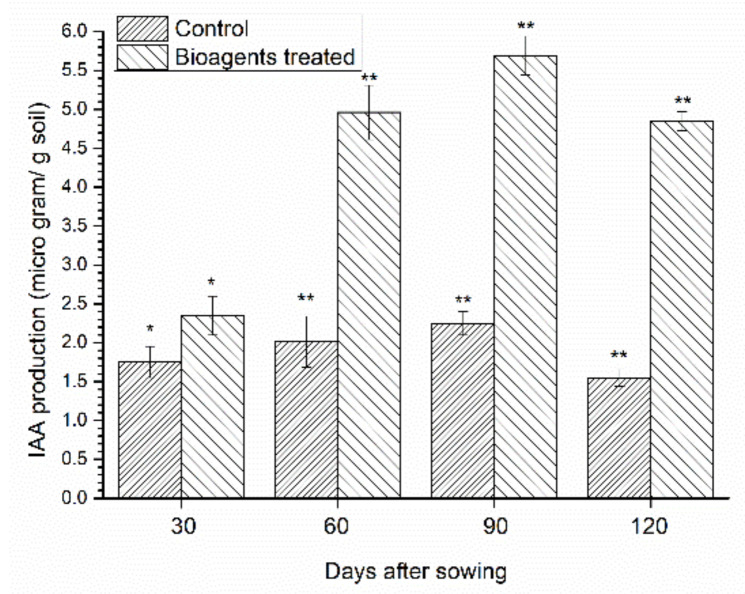
Effect of *T. harzianum* UBSTH-501 and *B. amyloliquefaciens* B-16 inoculation on IAA production in the wheat rhizosphere at 30, 60, 90, 120 DAS. Data are means and vertical bar lines represent standard deviation. Double asterisk (**) represents significant difference, while single asterisk (*) represents non-significant difference with their respective control.

A significant change in the IAA content was recorded in the rhizosphere of uninoculated control as well as in bioagents-inoculated plants under saline-sodic conditions at different time intervals and crop growth stages. Maximum IAA content was observed in the plants co-inoculated with *T. harzianum* UBSTH-501 and *B. amyloliquefaciens* B-16 at 90 DAS followed by 60 DAS. However, the least IAA content was recorded in uninoculated control at 120 DAS (Figure 1).

### 3.5. Effect of Microbial Inoculants on Plant Growth Promotion

A significant increase in plant height, number of tillers and total biomass accumulation (dry weight) was observed in plants co-inoculated with both the bioagents as compared to control at 30, 60, 90 and 120 DAS (Table 4). Total plant biomass varied considerably between the treatments, and it was highest (10.50 g) in plants co-inoculated with both the bioagents compared to control (6.88g) at 120 DAS. Similarly, plant height (cm) and the number of tillers plant per plant had the same pattern of differences as plant biomass (Table 4).

### 3.6. Effect of Microbial Inoculation on Membrane Thermostability, Chlorophyll Content, Accumulation of Biomolecules and Organic Solutes

**Figure 2:**
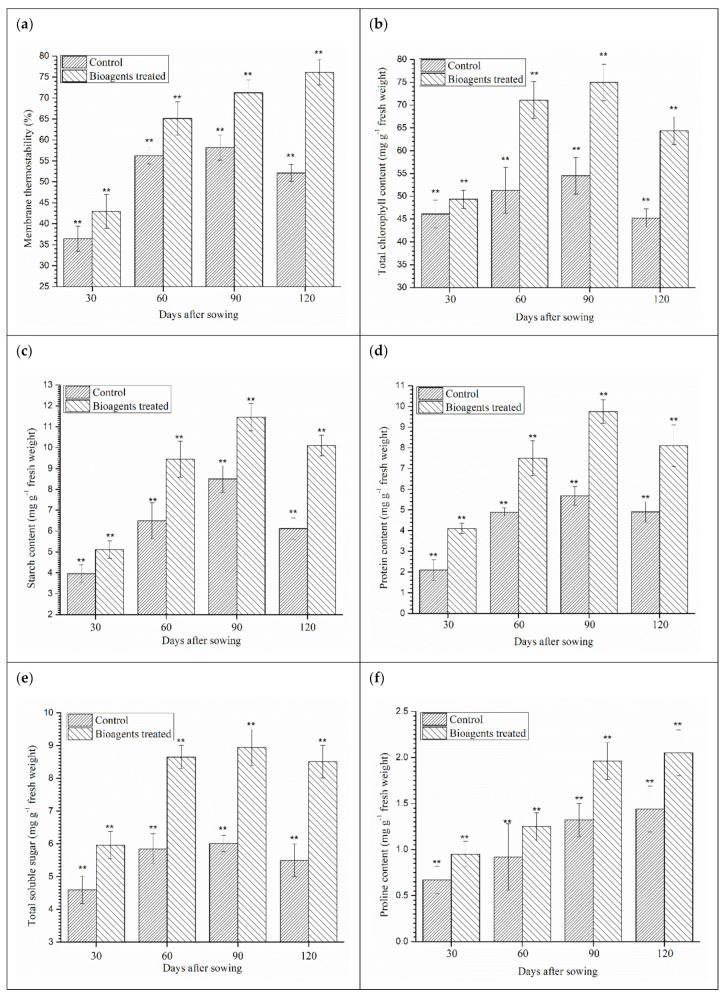
Effect of microbial inoculation on membrane thermostability, chlorophyll content, accumulation of biomolecules and organic solutes in wheat grown in saline sodic soil under field condition (**a**) membrane thermostability, (**b**) total chlorophyll content, (**c**) starch content, (**d**) total protein, (**e**) total soluble sugar, and (**f**) proline in wheat plants after 30, 60, 90, 120 days of sowing. Data are means and vertical bar lines represent standard deviation. Double asterisk (**) represents significant difference, while single asterisk (*) represents non-significant difference with their respective control.

The effect of microbial inoculation on membrane thermostability, chlorophyll content, accumulation of biomolecules and organic solutes was studied in the wheat crop grown in saline-sodic soils. Results showed that membrane thermostability was significantly higher in leaves of plants co-inoculated with both the bioagents as compared to the leaves of untreated control plants grown under salt-stressed conditions across the time interval (Figure 2a). Manifold increase in the total chlorophyll content was recorded in the co-inoculated plant leaves as compared to those of untreated control plants grown in saline-sodic soil at 30, 60, 90 and 120 DAS (Figure 2b).

The activation and accumulation of biomolecules related to salt tolerance were studied in the *B. amyloliquefaciens* B-16 and *T. harzianum* UBSTH-501 treated wheat crop grown in saline-sodic soil. Total starch, protein, soluble sugar and proline content in the leaves of wheat plants differed significantly in the treatments. The results showed that the plants treated with *B. amyloliquefaciens* B-16 and *T. harzianum* UBSTH-501 accumulated significantly higher amounts of total starch content (11.46 mg g^−1^) compared to the control plants (8.50 mg g^−1^) at 90 days of sowing, while being lowest at 30 days of sowing (Figure 2c). However, accumulation of total protein (Figure 2d), total soluble sugar (Figure 2e) and proline (Figure 2f) had the same pattern of differences as total starch content as compared to the untreated control plants at 30, 60, 90 and 120 DAS (Figure 2d–f).

### 3.7. Effect of Microbial Inoculation on Lipid Peroxidation and Antioxidant Enzymes

**Figure 3:**
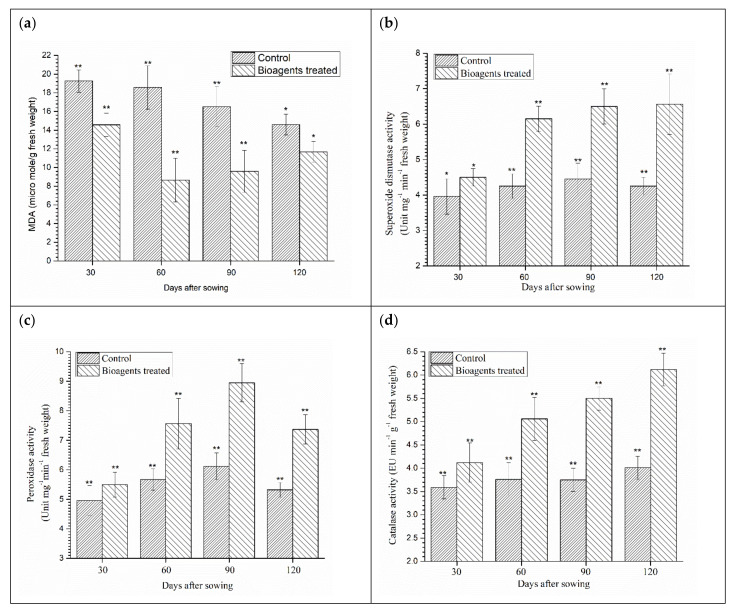
Effect of microbial inoculation on lipid peroxidation and antioxidant enzymes induced in wheat plants grown in saline sodic soil under field condition (**a**) lipid peroxidation (MDA), (**b**) superoxide dismutase, (**c**) peroxidase, and (**d**) catalase activity in wheat plants at 30, 60, 90, 120 DAS. Data are means and vertical bar lines represent standard deviation. Double asterisk (**) represents significant difference, while single asterisk (*) represents non-significant difference with their respective control.

In the presence of microbial inoculants, lipid peroxidation differed significantly. A significant reduction in malondialdehyde (MDA) was observed in plants subjected to salt stress irrespective of the microbial treatment (Figure 3a). Maximum reduction in the MDA was recorded in plants co-inoculated with *B. amyloliquefaciens* B-16 and *T. harzianum* UBSTH-501 at 60 DAS in comparison to the control plants grown under saline-sodic conditions. However, a significant increase in the said compound was observed in the untreated control plants (Figure 3a).

In comparison to the uninoculated control plants, a significant increase in terms of enzymatic activity was observed in the plants treated with both the bioagents at all the stages of crop growth (30, 60, 90 and 120 days of sowing). The highest SOD activity was recorded in plants treated with both the bioagents compared to the control (untreated) plants (Figure 3b). Moreover, the same treatment resulted in higher peroxidase (Figure 3c) and catalase (Figure 3d) activity in the plants co-inoculated with *B. amyloliquefaciens* B-16 and *T. harzianum* UBSTH-501 as compared to the untreated control (Figure 3c,d).

A direct correlation was recorded among the salt tolerance related biomolecules accumulation (Figure 2a–f), lipid peroxidation (Figure 3a), the activity of antioxidant enzymes (Figure 3b–d), seedling germination and vigour indices (Table 2), and growth attributes and biomass accumulation (Table 4) in plants co-inoculated with *B. amyloliquefaciens* B-16 and *T. harzianum* UBSTH-501 and grown under salt-stressed conditions at 30, 60, 90 and 120 days of sowing (Data not shown).

### 3.8. Expression Analyses of Zn Transporters and Biofortification of Wheat

**Figure 4:**
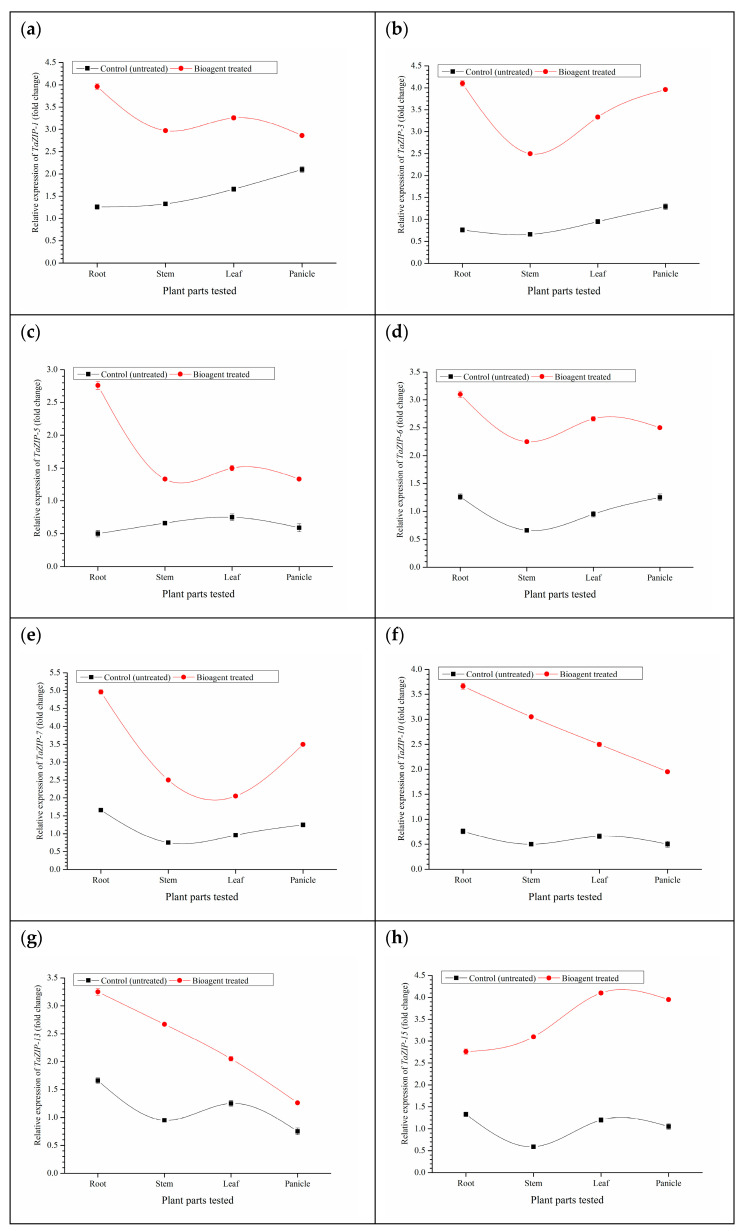
Effects of microbial inoculation on expression profile of ZIP transporter genes (fold change) in the wheat grown in saline sodic soil at 90 DAS, (**a**) *TaZIP*-*1*, (**b**) *TaZIP*-*3*, (**c**) *TaZIP*-*5*, (**d**) *TaZIP*-*6*, (**e**) *TaZIP*-*7*, (**f**) *TaZIP*-*10*, (**g**) *TaZIP*-*13*, and (**h**) *TaZIP-15.* Data are means and vertical bar represents standard deviation.

In general, microbial inoculation significantly up-regulates the *TaZIPs* in different parts of the plants such as root, stem, leaf and panicles as compared to untreated control plants at 90 DAS (grain filling stage). In the present investigation, eight key *TaZIP* transporter genes viz. *TaZIP*-*1*, *TaZIP*-*3*, *TaZIP*-*5*, *TaZIP*-*6*, *TaZIP*-*7*, *TaZIP*-*10*, *TaZIP*-*13*, and *TaZIP-15* were taken into consideration and observed differential expression across the plant parts (Figure 4). It was observed that the maximum expression of all the *TaZIP* transporter genes tested was recorded in the roots of microbial inoculated plants (2.76–4.96 folds) except for *TaZIP-15* as compared to untreated control plants (0.50–1.66 folds) grown under saline-sodic soil. Among the *TaZIP* transporter genes, the least expression was recorded in the *TaZIP*-*5* followed by *TaZIP*-*6* as compared to other *TaZIP* transporter genes. However, *TaZIP*-*1*, *TaZIP*-*3*, *TaZIP*-*7*, and *TaZIP*-10 were highly expressed genes in the wheat grown under saline-sodic soil. It was also observed that a significant expression of all these transporters were recorded in the panicles of plants co-inoculated with microbial inoculants at 90 DAS as compared to untreated control plants (Figure 4).

**Figure 5:**
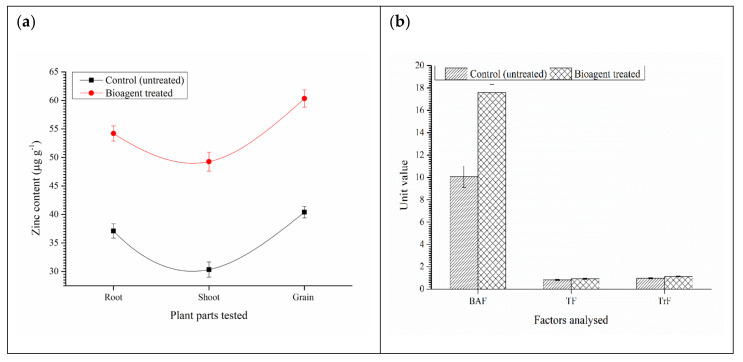
Effects of microbial inoculation on (**a**) Zn content in different parts of the wheat plants, and (**b**) Bioaccumulation factor, Translocation factor, and Transformation factor of the Zn in the wheat grown in saline sodic soil. Data are means and vertical bar represents standard deviation.

The Zn content was also estimated in the roots, shoots and grains of wheat co-inoculated with both the bioagents. Results revealed that significantly higher Zn was observed in the root (54.20 µg g^−1^), shoot (50.36 µg g^−1^) and grain (60.33 µg g^−1^) of the microbial inoculated plants as compared to untreated control (root: 37.10 µg g^−1^, shoot: 30.33 µg g^−1^ and grain: 36.39 µg g^−1^) (Figure 5a). Similarly, BAF (17.59), TF (0.925), and TrF (1.114) were also higher in the plants treated with both the bioagents as compared to untreated control (Figure 5b). These results corroborated the findings of expression analyses data.

### 3.9. Effect of Microbial Inoculation on Expression of TaHKT-1 and TaNHX-1 and Uptake of Na^+^, K^+^ and Ca^2+^

**Figure 6:**
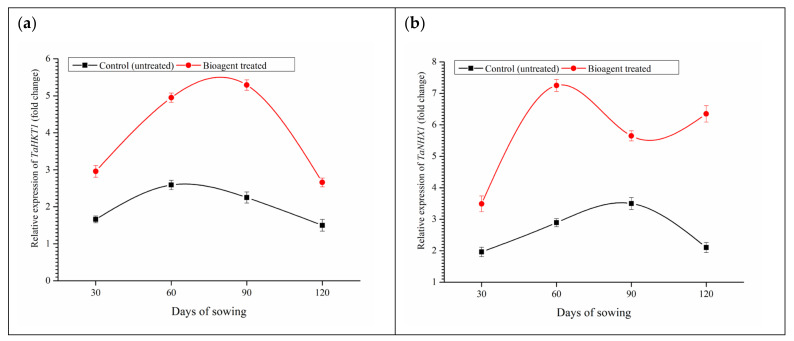
Effects of microbial inoculation on expression profile of (**a**) *TaHKT-1* and (**b**) *TaNHX-1* in the wheat grown in saline sodic soil at 90 DAS. Data are means and vertical bar represents standard deviation.

The expression profile of *TaHKT-1* and *TaNHX-1* was studied in the wheat roots grown in saline-sodic soil at 30, 60, 90, and 120 DAS. The maximum expression of *TaHKT-1* was recorded in the plants co-inoculated with both the bioagents at 90 DAS (5.29-folds) followed by 60 DAS (4.95-folds) when compared to untreated control plants (1.50–2.59 folds) grown in saline-sodic soil (Figure 6a). However, maximum expression of *TaNHX-1* was observed in the plants treated with both the bioagents at 60 DAS (7.25-folds) followed by 120 DAS (6.35-folds) and 90 DAS (5.65-folds) when compared to untreated control plants (1.96–3.50 folds) grown in saline-sodic soil (Figure 6b).

High salt concentration in soil colloid significantly increased Na^+^ ion concentration and decreased K^+^ and Ca^2+^ concentration in plants roots and shoots of control plants. Results showed that co-inoculation with *B. amyloliquefaciens* B-16 and *T. harzianum* UBSTH-501 resulted in a decrease in Na^+^ uptake and an increase in K^+^ and Ca^2+^ uptake in plants. There was a substantial decrease in Na^+^ uptake in the plants treated with bioagents as compared to the respective untreated controls at different time intervals. However, the maximum increase in K^+^ and Ca^2+^ uptake was recorded in wheat plants co-inoculated with both the bioagents (13.09 and 11.44 kg ha^−1^, respectively) as compared to the untreated control plants (6.50 and 7.00 kg ha^−1^, respectively) at 120 DAS (Table 5).

### 3.10. Effect of Microbial Inoculation on Yield and Yield Attributing Traits

**Figure 7:**
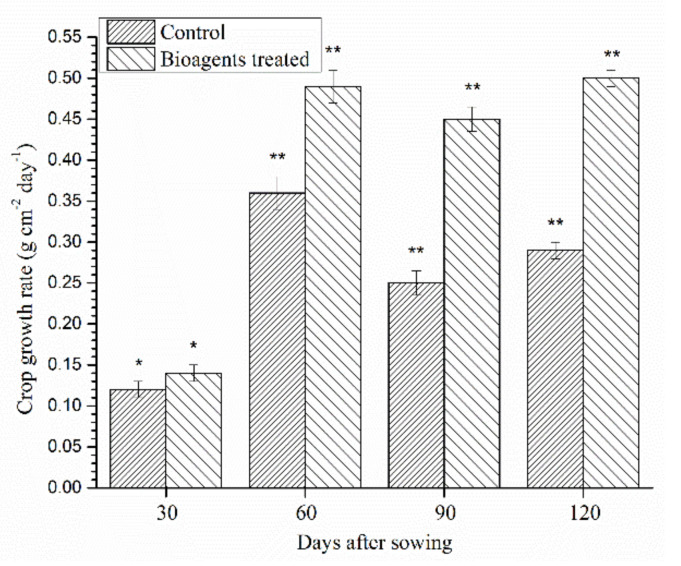
Effect of microbial inoculation on crop growth rate (CGR) in wheat grown in saline sodic soil under field condition at 30, 60, 90, 120 DAS. Data are means and vertical bar lines represent standard deviation. Double asterisk (**) represents significant difference, while single asterisk (*) represents non-significant difference with their respective control.

Data on crop growth rate (g cm^−2^ day^−1^) indicated that treatment with both the bioagents enhanced shoot length and biomass accumulation significantly (*p* < 0.05) as compared to control plants at 60, 90 and 120 DAS. In contrast, crop growth rate did not differ significantly between bioagents treated and control plants at 30 days of sowing (Figure 7).

Results of the present investigation indicate that treatment with bioagents significantly (*p* < 0.05) increased the yield and yield attributing traits of wheat crops grown in saline-sodic soil. A considerable increase in yield attributing traits was observed in plants co-inoculated with the bioagents as compared to the control plants (Table 6). Bioagents inoculation improved the spike length and number of seeds per spike by 23.07% and 13.16%, respectively in comparison to the untreated control. A similar trend was also observed in other yield attributing traits. The highest increase in total grain yield was recorded in plants treated with both the bioagents (27.20 q ha^−1^) compared to their control (22.80 q ha^−1^) under salt stress conditions (Table 6).

## 4. Discussion

Soil salinity and sodicity have become important factors that reduce the availability of cultivable land affecting agricultural production worldwide and together, are predicted to become a larger part of the problem in the times to come [3,38]. Globally, soil salinization reduces the available arable land in terms of the gross cropped area by 1–2% annually in the arid and semi-arid regions [61]. Salt stress affects plant growth and yield in many crop species including cereals (wheat, rice and maize), forages (clover), pulse crops (pea, chickpea, pigeonpea) and horticultural crops (potato and tomato) [62,63,64]. They are relatively more susceptible to excessive salt concentration. The present study demonstrates the effects of salt-tolerant *T. harzianum* UBSTH-501 and *B. amyloliquefaciens* B-16 and attempts have been made to decipher the mechanisms of salt tolerance in the wheat plants and their impact on growth, yield and physiological traits of wheat crops grown in saline-sodic soil. The selected strains were screened for salt tolerance and PGP traits. The selected strains were able to tolerate salt concentration (NaCl) up to 5% and were found positive for different PGP traits including solubilisation of Zn. The findings of the present study established that, under salt stress conditions, germination percent and vigour indices of wheat increased following the treatment with bioagents as compared to the untreated control. Extensive research has been conducted to demonstrate the beneficial effects of salt-tolerant plant growth promoting microorganisms (PGPMs) on plant growth [29,37,38,65,66,67,68]. Plants challenged with high salt stress tend to overproduce and accumulate biomolecules/organic solutes and other high molecular weight compounds (osmolytes and compatible organic solutes) in their tissues to combat stress [28,69,70]. Plants treated with bioagents under salt stress conditions reprogrammed the catabolic and metabolic cascade/network related to salt tolerance and signalling at the cellular level. Indole acetic acid (IAA) is a phytohormone, involved in root initiation, cell enlargement and cell division; therefore, the IAA production by salt-tolerant PGPMs is crucial for plant growth. The results of this investigation corroborate the fact that a significant amount of IAA is synthesized and found in the rhizospheric soil of bioagents-treated plants grown in saline-sodic soil as compared to the control (Figure 1). A significant increase in plant growth and total plant biomass was recorded in the plants treated with bioagents. These results are in agreement with the findings of Wang et al. [71], who demonstrated that application of *B. amyloliquefaciens* promotes plant growth by synthesizing plant growth hormones, such as indole-3-acetic acid (IAA), cytokinin and gibberellins; reduction in the volatile plant hormone, ethylene by the production of 1-amino cyclopropane-1-carboxylate (ACC) deaminase and through increased uptake of nutrients from the soil [24,37,38,72,73,74].

IAA in the rhizosphere significantly increases root growth which constitutes a greater root surface area that enables the plant to obtain more nutrients from the soil [75,76]. High salt concentration adversely affects the microbial activity in the rhizosphere [77]. The findings of the present investigation establish a direct correlation between salt concentration and microbial population in the rhizosphere. Maximum microbial population was encountered in the rhizosphere of wheat plants treated with bioagents after 60 and 90 DAS. Even a small quantity of organic matter applied with the *T. harzianum* as a carrier material attracted microbes towards the rhizosphere. Results also demonstrated that the application of bioagents modulated the root secretion system and pH of the rhizosphere environment which got reduced up to some extent (Data not shown). It was hypothesized that these microbes may balance the equilibrium of certain cations in the rhizosphere and modulate the uptake and translocation system in the plants. Similar observations were also reported by Paul and Lade [35], who demonstrated that microbes could alter the uptake of toxic ions and nutrients by altering host physiology or by directly reducing uptake and accumulation of Na^+^ and Cl^−^, while increasing the uptake and translocation of other cations, including zinc, which is evidenced from Zn concentration in the root, shoot and grains. However, the exact mechanisms are still unknown [25,76]. The ZIP (Zn-regulated, iron-regulated transporter-like protein) transporter is one of the widely studied key gene families regulating the uptake, and transport of Zn and Fe across the plant kingdom [78,79,80,81]. The ZIP transporters have been widely and perhaps systematically studied in *Arabidopsis* and several other plant species. However, the role of ZIP transporters in wheat upon inoculation of *T. harzianum* and *B. amyloliquefaciens* and grown under saline-sodic conditions is not well understood at present and needs in-depth investigation. In the present investigation, eight key *TaZIP* transporter genes viz. *TaZIP*-*1*, *TaZIP*-*3*, *TaZIP*-*5*, *TaZIP*-*6*, *TaZIP*-*7*, *TaZIP*-*10*, *TaZIP*-*13*, and *TaZIP-15* were taken into consideration. Differential expression of these genes was observed across the plant parts (Figure 4). Significantly higher expression of all the *TaZIP* transporter genes were recorded in the roots of microbial inoculated plants (2.76–4.96 folds) except for *TaZIP-15* as compared to untreated control plants. Gene expression results clearly indicated that microbial inoculants modulated the expression profile of *TaZIP* transporter directly and/or indirectly. Enhanced uptake and translocation of Zn in roots and shoots of the plants inoculated with *T. harzianum* and *B. amyloliquefaciens* as against absolute control plants is in line with the previous reports [14,82,83]. Ramesh et al. [14] reported that *Bacillus aryabhattai* solubilized the insoluble Zn present in the soil and made it available to the wheat and soybean plants. It was also reported that the application of PGPMs enhanced the translocation of Zn towards wheat grains through induction of physiological processes, mineralization and solubilization of Zn in the rhizosphere [84,85,86,87].

As the salt level increases in the soil, Na^+^ exerts ionic competence diminishing the ability of ion uptake by plants. The high level of Na^+^ in the plants affects major metabolic processes such as photosynthesis, protein synthesis and energy and lipid metabolism [9,10,88,89]. Photosynthetic capacity is also reduced under high salt stress due to the osmotic stress and cause partial closure of stomata [89,90]. Plants can also suffer from membrane destabilisation and in general nutrient imbalance [89,91]. Our results also established that plants accumulate high molecular weight osmolytes such as proline and other organic solutes in stress conditions [29,92]. The accumulation of osmolytes and organic solutes significantly increased in the co-inoculated plants as compared to the control (Figure 3a–f). Qurashi and Sabri [65] stated that endogenous osmolytes such as proline, glycine betaine, sugars and choline are accumulated in moderately halophilic bacterial strains, *S. haemolyticus* and *B. subtilis* isolated from chickpea rhizosphere. These osmolytes improve the growth of bacteria as well as plants by alleviating salt stress. The formation of ROS upon salt shock in plants may cause damage to lipids, protein and nucleic acids. Generally, ROS production is favoured due to the over-reduction of the photosynthetic electron chain by limiting the rate of photosynthesis under high salt stress [93,94,95,96,97,98]. PGPMs-mediated induction of stress tolerance in plants is well-acknowledged. Results of the present investigation revealed that the application of bioagents in wheat induced the cascades/pathways responsible for the synthesis of antioxidant enzymes and lipid peroxidation. In the present study, the enzymatic activities increased significantly after bioagents inoculation in comparison to the control plants. Paul and Lade [35] reported that activities of antioxidant enzymes generally increase when plants are subjected to biotic or abiotic stress. Antioxidant enzymes (superoxide dismutase and peroxidase) are generally involved in eliminating reactive oxygen species [98,99,100,101,102], reducing lipid peroxidation [102] and increasing membrane thermostability [35]. Excess sodium and, more importantly, chloride in the plant cell has the potential to affect plant metabolism and cause cell swelling and physiological changes resulting in reduced energy production [101,103].

In the present study, bioagents inoculation showed a significant increase in the expression of *TaHKT-1* and *TaNHX-1.* uptake and translocation of K^+^ and Ca^2+^ in wheat as compared to the control across the growth stages resulting in increased ion equilibrium at the cellular level. Likewise, a manifold increase in the growth attributes of plants treated with bioagents under salt stress conditions was recorded. This equilibrium might directly or indirectly help in plant growth and development under salt stress conditions. Under high salt stress, excessive Na^+^ and Cl^−^ concentration in the rhizosphere soil induce competitive interactions with other nutrients/ions including K^+^, NO^3−^ and H_2_PO_4_^−^ [38,101]. It impaired the structure and function of binding sites and transport proteins in root epidermal cells which affect the downward signalling and thereafter affect the translocation, deposition and partitioning of essential nutrients including Zn in the plant tissues [98,101,104,105]. An increase in the uptake of Na^+^ or a decrease in the uptake of Ca^2+^ and K^+^ in leaves leads to nutritional imbalances. Accumulation of excessive Na^+^ may cause metabolic disturbances in processes where low Na^+^ and high K^+^ or Ca^2+^ are required for optimum function [106,107]. Once the capacity of cells to store salts is exhausted, salts start building up in the intercellular spaces leading to exo-osmosis, cell dehydration and death [25,108].

**Figure 8:**
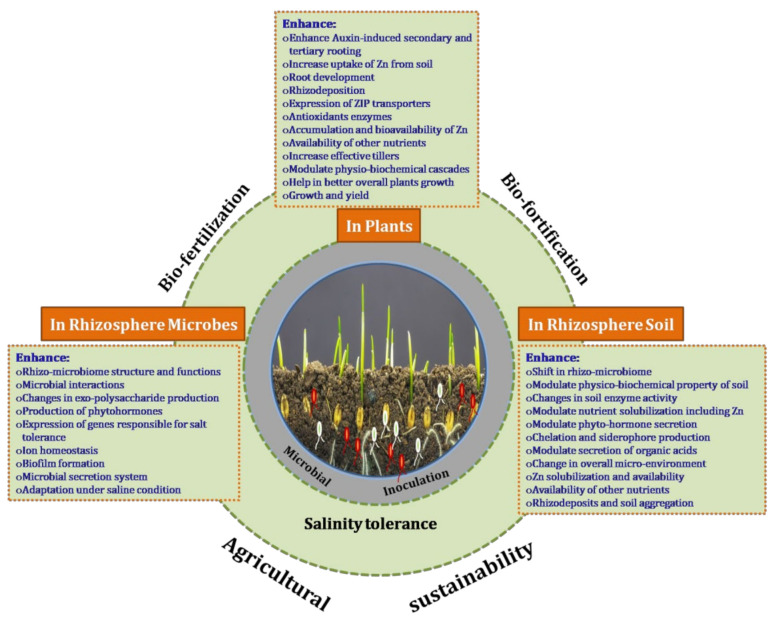
Schematic diagram representing holistic overview of microbial inoculation, *T. harzianum* and *B. amyloliquefaciens* on rhizosphere microbes, plants and rhizosphere soil at different crop growth stages.

Salt stress in plants is a cumulative effect of osmotic and ionic stresses which negatively affects plant growth and yield. Researchers have shown that multiple genes are involved in the salt tolerance mechanism in several plant species [70,109,110]. They have been reported to be involved in signal transduction, ion transporters, transcription regulation and metabolic pathways [37,38,111,112]. Among them, some are constitutive, and others are inducible. PGPMs colonise the rhizosphere of plants and promote plant growth through various means (Figure 8). Zhang et al. [34] reported that inoculation with *B. subtilis* GB03 regulated the potassium transporter *HKT1* gene in *Arabidopsis thaliana* and thereby increased the level of salt tolerance in plants [113,114,115]. The investigation revealed that certain volatiles emitted by PGPMs down-regulate *HKT1* expression in roots but up-regulate it in shoots, leading to lower Na^+^ levels and recirculation of Na^+^ in the whole plant under salt-stressed conditions [34]. These results sustained the idea that bacteria can mediate the expression of high-affinity K^+^ transporter in *Arabidopsis* (*AtHKT1*) under saline conditions [116].

Salinity and sodicity play a vital role in the availability of Zn in soil and its translocation to the plants [17,117]. The results of the present investigation indicated that microbial inoculants gave good results in controlling seedling mortality and improved Zn uptake and translocation in wheat under field experiment. Further, microbial inoculants significantly affect the expression profile of Zn transporter (*TaZIP*) genes in the wheat grown in saline-sodic soil. These transporters play a crucial role in uptake of Zn from soil and translocate to other parts of the plants. It was observed that microbial inoculants successfully up regulated the ZIP transporter genes thereby increasing uptake and translocation of Zn in wheat even under salt stressed conditions. These results are in agreement with the several other researchers [13,118,119,120,121]. However, further pieces of evidence are needed to verify the exact role of these bioagents along with other putative mechanisms participating in microbe-mediated salinity tolerance in wheat crop.

## 5. Conclusions

In this study, we have shown that compatible salt-tolerant *T. harzianum* UBSTH-501 and *B. amyloliquefaciens* B-16 with multiple PGP traits can enhance plant growth, Zn-biofortification and yield under salt-stressed conditions. The results confirmed that co-inoculation of these two bioagents increases the microbial population in the rhizosphere soil that might contribute to greater ionic balance under higher salt concentrations. Our findings focus on microbe-mediated induction and accumulation of osmolytes and organic solutes, synthesis of phytohormones, antioxidant enzymes to induce membrane thermostability and metabolic function at cellular levels. Along with salt tolerance, these microbial inoculants enhanced the expression of ZIP transporter genes and thereby increased the uptake and translocation of Zn. This Zn pool is further translocated into the grains and improves the nutritional quality of biofortified wheat. Hence, the use of these multi-traits linked salt-tolerant bioagents as bioinoculant holds great potential to combat salt stress in wheat crops cultivated in saline-sodic of soils of eastern Uttar Pradesh of India.

## Figures and Tables

**Table 1:** Oligonucleotide primer sequences used for SYBR Green real time qPCR expression analysis.

S.No.	Gene Symbol	Forward Primer	Reverse Primer
(A) Zn transporter (*TaZIP*) genes			
1.	*TaZIP1*	GTCCCCCTACTTCTACCGCT	TGGTTGACCCTCTGCCTGTT
2.	*TaZIP3*	GGGAAATGGAGAACYCCTGGATG	GGCATAGAGATCTTGAAAGCAATTGC
3.	*TaZIP5*	AGGTTTCGCCTCAAGTCTGTCTTG	GGCTATTCTCGTCGTAAGCAGAG
4.	*TaZIP6*	GTCATCATCTCTGAAACTGAAGAAGG	CCCTCTATACATTTCACTATGRCC
5.	*TaZIP7*	ACAGGCAGTATGTTSGGACGTAG	CAGCAAGTGATGGCCTATGTCG
6.	*TaZIP10*	GTGGATCTCATTGCTGCTGA	AGCCCAAATAGCCAGTGATG
7.	*TaZIP13*	CGCAAGCSTACAACATGAAACAGT	CTTYAGACACGCTACTGGGTTGG
8.	*TaZIP13*	CGCGAGCCTACAACTTGAAACAG	CTTYAGACACGCTACTGGGTTGG
9.	*TaZIP15*	CTCTCTGCGCTGGTTGCTTT	TGGGAGGACTCCGGCAACAG
(B) Salinity stress related genes			
10.	*TaHKT*-1	CAAAGGTGAAGGAGCTGAGG	GAGCTGAGCCCATCAAAGAC
11.	*TaNHX*-1	GAATGCCACTCAGATCCAGC	GCTGCTGGGTGGCTTAGTGC
(C) Housekeeping genes			
12.	*TaActin3*	GACGCACAACAGGTATCGTGTTG	CAGCGAGGTCAAGACGAAGGATG
13.	*TaSuccDH*	TTTGCTCTCCGTGGTGCCTTTGG	GAAGATGTGTAGCTCCTTGCTTGC

**Table 2:** Effect of bioagents on germination and vigour of wheat grown under salt stress conditions 30 days after sowing.

Treatments	Germination (%)	VigourIndex I	VigourIndex II
*T. harzianum* UBSTH-501	87.23 ± 2.25 a	3413.22 ± 10.21 a	1.95 ± 0.45 a
*B. amyloliquefaciens* B-16	85.26 ± 2.50 b	3324.16 ± 9.26 b	1.87 ±0.25 a
Control (untreated)	80.92 ± 3.52 c	3112.25± 8.20 c	1.75 ± 0.25 b

Data are means ± SEM, values in column followed by the different letter are significantly different at *p* < 0.05.

**Table 3:** Effect of bioagent treatment and agronomic practices of log CFU count of bacteria, fungi and actinomycetes in wheat rhizosphere under salt stress conditions.

Treatments	Fungi				Bacteria				Actinomycetes			
	30 DAS ^†^	60 DAS	90 DAS	120 DAS	30 DAS	60 DAS	90 DAS	120 DAS	30 DAS	60 DAS	90 DAS	120 DAS
Control (untreated)	2.02 ± 0.25 b	3.01 ± 0.15 a	2.92 ± 0.50 b	2.62 ± 0.57 b	2.15 ± 0.50 b	3.46 ± 0.16 b	3.92 ± 0.15 b	3.67 ± 0.25 b	1.61 ± 0.10 a	1.92 ± 0.15 b	2.01 ± 0.33 b	1.72 ± 0.11 b
Bioagents treated	2.85 ± 0.33 a	3.01 ± 0.36 a	3.96 ± 0.33 a	3.30 ± 0.66 a	3.56 ± 0.20 a	6.96 ± 0.66 a	7.12 ± 1.15 a	4.92 ± 0.50 a	1.88 ± 0.33 a	2.67 ± 0.20 a	2.46 ± 0.45 a	2.42 ± 0.25 a

^†^ DAS represent days after sowing, data are means ± SEM, values within a column followed by the same letter are not significantly different at *p* < 0.05.

**Table 4:** Effect of bioagents on different growth parameters of wheat plants grown under salt stress conditions.

Treatments	Plant Height (cm)				Number of Tillers Plant^−1^				Plant Biomass on Dry wt. Basis (g)			
	30 DAS ^†^	60 DAS	90 DAS	120 DAS	30 DAS	60 DAS	90 DAS	120 DAS	30 DAS	60 DAS	90 DAS	120 DAS
Control (untreated)	17.50 ± 1.02 b	38.60 ± 2.25 b	70.30 ± 2.50 b	75.05 ± 1.57 b	2.05 ± 0.55 b	4.30 ± 0.36 b	4.90 ± 0.25 b	4.96 ± 0.45 b	1.89 ± 0.22 b	4.80 ± 0.50 b	5.21 ± 0.33 b	6.88 ± 0.21 b
Bioagents treated	24.70 ± 2.01 a	61.90 ± 2.36 a	95.40 ± 3.50 a	100.30 ± 2.65 a	3.25 ± 0.22 a	8.40 ± 1.02 a	8.90 ± 0.66 a	8.90 ± 0.40 a	2.35 ± 0.11 a	6.54 ± 0.25 a	8.20 ± 0.25 a	10.50 ± 0.75 a

^†^ DAS represent days after sowing, data are means ± SD, values within a column followed by the same letter are not significantly different at *p* < 0.05.

**Table 5:** Effect of bioagents on Na^+^, K^+^ and Ca^2+^ uptake in plants (kg ha^−1^) grown under salt stress conditions.

Treatments	Na^+^ Uptake				K^+^ Uptake				Ca^2+^ Uptake			
	30 DAS ^†^	60 DAS	90 DAS	120 DAS	30 DAS	60 DAS	90 DAS	120 DAS	30 DAS	60 DAS	90 DAS	120 DAS
Control (untreated)	0.44 ± 0.01 a	3.06 ± 0.25 a	5.93 ± 0.55 a	8.68 ± 1.02 a	0.58 ± 0.01 b	2.36 ± 0.20 b	3.90 ± 0.66 b	6.50 ± 0.80 b	0.45 ± 0.01 a	2.20 ± 0.19 b	3.77 ± 0.45 b	7.00 ± 0.95 b
Bioagents treated	0.54 ± 0.05 a	2.82 ± 0.33 b	5.71 ± 0.82 b	7.10 ± 0.66 b	1.06 ± 0.15 a	5.54 ± 0.55 a	9.36 ± 1.33 a	13.09 ± 1.66 a	0.75 ± 0.01 a	4.25 ± 0.25 a	7.86 ± 0.75 a	11.44 ± 4.25 a

^†^ DAS represent days after sowing, data are means ± SD, values within a column followed by the same letter are not significantly different at *p* < 0.05.

**Table 6:** Effect of bioagents on yield and yield attributes of wheat grown under salt stress conditions at harvest.

Parameters	Control (Untreated)	Bioagents Treated
Spike length (cm)	9.10 ± 1.02 b	11.20 ± 1.15 a
Spike weight (g)	1.66 ± 0.20 b	2.10 ± 0.21 a
Spikelet spike ^−1^	17.05 ± 1.25 b	19.50 ± 1.45 a
Number of seeds spike ^−1^	44.80 ± 2.01 b	50.70 ± 3.02 a
Seed weight (g) spike ^−1^	1.50 ± 0.11 b	1.70 ± 0.33 a
Test weight (g)	37.30 ± 1.33 b	39.20 ± 1.36 a
Seed yield (q ha ^−1^)	22.80 ± 1.03 b	27.20 ± 1.25 a
Straw yield (q ha ^−1^)	33.20 ± 1.25 b	41.70 ± 1.54 a
Biological yield (q ha ^−1^)	56.00 ± 2.25 b	68.90 ± 2.66 a

Data are means ± SD, values within a row followed by the same letter are not significantly different at *p* < 0.05.

## Data Availability

Not applicable.

## Supplementary Materials

The following are available online at https://www.mdpi.com/article/10.3390/ijerph18189936/s1, Table S1: Physico-biochemical properties of initial soil used in this study, Table S2: Salt tolerance (NaCl) and biochemical properties of selected strains used in this study.

## References

[1] Flowers T.J., Garcia A., Koyama M., Yeo A.R. (1997). Breeding for salt tolerance in crop plants—the role of molecular biology. Acta Physiol..

[2] Szabolcs I., Pessarakali M. (1994). Soils and salinisation. Handbook of Plant and Crop Stress.

[3] Das D.K. (2009). Introductory Soil Science.

[4] National Remote Sensing Application and Associates (1996). Mapping of Salt Affected Soils of India. 1:250000 Map Sheets, Legend.

[5] Zhang S., Gan Y., Xu B. (2016). Application of plant-growth-promoting fungi *Trichoderma longibrachiatum* T6 enhances tolerance of wheat to salt stress through improvement of antioxidative defense system and gene expression. Front. Plant Sci..

[6] Liu S.H., Kang Y.H. (2014). Changes of soil microbial characteristics in saline-sodic soils under drip irrigation. J. Soil Sci. Plant Nutr..

[7] Wong V.N., Greene R.S., Dalal R.C., Murphy B.W. (2010). Soil carbon dynamics in saline and sodic soils: A review. Soil Use Manag..

[8] Dargan K.S., Gaul B.L. (1974). For paddy in alkali soil seedling age and planting date vital. Indian Farming.

[9] Mishra N., Khan S.S., Sundari S.K. (2016). Native isolate of *Trichoderma*: A biocontrol agent with unique stress tolerance properties. World J. Microbiol. Biotechnol..

[10] Kumar K., Manigundan K., Amaresan N. (2017). Influence of salt tolerant *Trichoderma* spp. on growth of maize (*Zea mays* L.) under different salinity conditions. J. Basic Microbiol..

[11] Liu X., Zhao H., Chen S. (2006). Colonization of maize and rice plants by strain Bacillus megateriumC4. Curr. Microbiol..

[12] Aravind P., Prasad M.N.V. (2004). Zinc protects chloroplasts and associated photochemical functions in cadmium exposed Ceratophyllumdemersum L., a fresh water macrophyte. Plant Sci..

[13] Ramesh A., Sharma S.K., Sharma M.P., Yadav N., Joshi O.P. (2014). Inoculation of zinc solubilizing *Bacillus aryabhattai* strains for improved growth, mobilization and biofortification of zinc in soybean and wheat cultivated in Vertisols of central India. Appl. Soil Ecol..

[14] Gunes A., Inal A., Alpaslan M. (1996). Effect of salinity on stomatal resistance, proline and mineral composition of pepper. J. Plant Nutr..

[15] Jamalomidi M., Esfahani M., Carapetian J. (2006). Zinc and salinity interaction on agronomical traits, chlorophyll and proline content in lowland rice (*Oryza sativa* L.) genotypes. Pak. J. Biol. Sci..

[16] Khoshgoftarmanesh A.H., Shariatmadari H., Karimian N., Khajehpour M.R. (2006). Responses of wheat genotypes to zinc fertilization under saline soil conditions. J. Plant Nutr..

[17] Tinker P.B., Lauchli A. (1984). Advances in Plant Nutrition.

[18] Genc Y., McDonald G.K., Graham R.D., Li C.J., Zhang F.-S., Doberman A., Hinsinger P., Lambers H., Li X.L., Marschner P., Maene L., McGrath S.P., Oenema O. (2005). The interactive effects of zinc and salt on growth of wheat. Plant Nutrition for Food Security, Human Health and Environmental Protection.

[19] Alpaslan M., Inal A., Gunes A., Çikili Y., Ozcan H. (1999). Effect of zinc treatment on the alleviation of sodium and chloride injury in tomato (*Lycopersicum esculentum* (L.) Mill. cv. Lale) grown under salinity. Turk. J. Agric. For..

[20] Cakmak I. (2000). Possible roles of zinc in protecting plant cells from damage by reactive oxygen species. New Phytol..

[21] Joshi A.K., Kumari M., Singh V.P., Reddy C.M., Kumar S., Rane J., Chand R. (2007). Stay green trait: Variation, inheritance and its association with spot blotch resistance in spring wheat (*Triticum aestivum* L.). Euphytica.

[22] Joshi A.K., Mishra B., Chatrath R., Ferrara G.O., Singh R.P. (2007). Wheat improvement in India: Present status, emerging challenges and future prospects. Euphytica.

[23] Joshi A.K., Chand R. (2011). Progress of researches done to understand host-pathogen relationship for spot blotch pathogen of wheat. J. Wheat Res..

[24] Singh C., Singh P., Singh R. (2008). Modern Techniques of Raising Field Crops.

[25] Singh R.P., Jha P., Jha P.N. (2015). The plant-growth-promoting bacterium *Klebsiella* sp. SBP-8 confers induced systemic tolerance in wheat (*Triticum aestivum)* under salt stress. J. Plant Physiol..

[26] Singh Y.P., Mishra V.K., Singh S., Sharma D.K., Singh D., Singh U.S., Singh R.K., Haefele S.M., Ismail A.M. (2016). Productivity of sodic soils can be enhanced through the use of salt tolerant rice varieties and proper agronomic practices. Field Crops Res..

[27] Ebel C., BenFeki A., Hanin M., Solano R., Chini A. (2018). Characterization of wheat (*Triticum aestivum*) TIFY family and role of *Triticum Durum* TdTIFY11a in salt stress tolerance. PLoS ONE.

[28] Abhinandan K., Skori L., Stanic M., Hickerson N.M.N., Jamshed M., Samuel M.A. (2018). Abiotic stress signaling in wheat–an inclusive overview of hormonal interactions during abiotic stress responses in wheat. Front. Plant Sci..

[29] Singh U.B., Malviya D., Wasiullah, Singh S., Imran M., Pathak N., Alam M., Rai J.P., Singh R.K., Sarma B.K. (2016). Compatible salt-tolerant rhizosphere microbe-mediated induction of phenylpropanoid cascade and induced systemic responses against *Bipolaris sorokiniana* (Sacc.) Shoemaker causing spot blotch disease in wheat (*Triticum aestivum* L.). Appl. Soil Ecol..

[30] Siddaiah C.N., Satyanarayana N.R., Mudili V., Gupta V.K., Gurunathan S., Rangappa S., Huntrike S.S., Srivastava R.K. (2017). Elicitation of resistance and associated defense responses in *Trichoderma hamatum* induced protection against pearl millet downy mildew pathogen. Sci. Rep..

[31] Glick B.R., Cheng Z., Czarny J., Duan J. (2007). Promotion of plant growth by ACC deaminase-producing soil bacteria. Eur. J. Plant Pathol..

[32] Orhan F. (2016). Alleviation of salt stress by halotolerant and halophilic plant growth-promoting bacteria in wheat (*Triticum aestivum*). Braz. J. Microbiol..

[33] Santoyo G., Moreno-Hagelsieb G., del Carmen Orozco-Mosqueda M., Glick B.R. (2016). Plant growth-promoting bacterial endophytes. Microbiol. Res..

[34] Zhang H., Kim M.S., Sun Y., Dowd S.E., Shi H., Paré P.W. (2008). Soil bacteria confer plant salt tolerance by tissue-specific regulation of the sodium transporter HKT1. Mol. Plant Microbe Interact..

[35] Paul D., Lade H. (2014). Plant-growth-promoting rhizobacteria to improve crop growth in saline soils: A review. Agron. Sustain. Dev..

[36] Shrivastava P., Kumar R. (2015). Soil salinity: A serious environmental issue and plant growth promoting bacteria as one of the tools for its alleviation. Saudi J. Boil. Sci..

[37] Singh S., Singh U.B., Trivedi M., Sahu P.K., Paul S., Paul D., Saxena A.K. (2019). Seed biopriming with salt-tolerant endophytic *Pseudomonas geniculata*-modulated biochemical responses provide ecological fitness in maize (*Zea mays* L.) grown in saline sodic soil. Int. J. Environ. Res. Public Health.

[38] Singh S., Singh U.B., Trivdi M., Malviya D., Sahu P.K., Roy M., Sharma P.K., Singh H.V., Manna M.C., Saxena A.K. (2021). Restructuring the cellular responses: Connecting microbial intervention with ecological fitness and adaptiveness to the maize (*Zea mays* L.) grown in saline–sodic soil. Front. Microbiol..

[39] Horie T., Sugawara M., Okunou K., Nakayama H., Schroeder J.I., Shinmyo A., Yoshida K. (2008). Functions of HKT transporters in sodium transport in roots and in protecting leaves from salinity stress. Plant Biotech..

[40] Brick J.M., Bostock R.M., Silversone S.E. (1991). Rapid in situ assay for indole acetic acid production by bacteria immobilized on a nitrocellulose membrane. Appl. Environ. Microbiol..

[41] Schwyn B., Neilands J.B. (1987). Universal chemical assay for the detection and determination of siderophores. Anal. Biochem..

[42] Dey R.K., Pal K.K., Bhatt D.M., Chauhan S.M. (2004). Growth promotion and yield enhancement of peanut (*Arachis hypogaea* L.) by application of plant growth-promoting rhizobacteria. Microbiol. Res..

[43] Gaur A.C. (1990). Physiological Functions of Phosphate Solubilizing Micro-Organisms. Phosphate Solubilizing Micro-Organisms as Biofertilizers.

[44] Boller T., Mauch F. (1988). Colorimetric assay for chitinase. Methods Enzymol..

[45] Whitman W.B., Goodfellow M., Kämpfer P., Busse H.-J., Trujillo M.E., Ludwig W., Suzuki K.-i. (2012). Bergey’s Manual of Systematic Bacteriology Parts A and B.

[46] Singh U.B., Malviya D., Wasiullah, Singh S., Pradhan J.K., Singh B.P., Roy M., Imram M., Pathak N., Baisyal B.M. (2016). Bio-protective microbial agents from rhizosphere eco-systems triggering plant defense responses provide protection against sheath blight disease in rice (*Oryza sativa* L.). Microbiol. Res..

[47] Singh U.B., Malviya D., Khan W., Singh S., Karthikeyan N., Imran M., Rai J.P., Sarma B.K., Manna M.C., Chaurasia R. (2018). Earthworm grazed-*Trichoderma harzianum* biofortified spent mushroom substrates modulate accumulation of natural antioxidants and bio-fortification of mineral nutrients in tomato. Front. Plant Sci..

[48] ISTA Seed Testing International, ISTA Secretariat, CH – Switzerland, ISTA News Bulletin No. 126, 2003. https://www.seedtest.org/upload/cms/user/STI126.

[49] Udayashankar A.C., Nayaka S.C., Reddy M.S., Srinivas C. (2011). Plant growth-promoting rhizobacteria mediate induced systemic resistance in rice against bacterial leaf blight caused by *Xanthomonas oryzae* pv. *oryzae*. Biol. Control..

[50] Abdul-Baki A.A., Anderson J.D. (1973). Vigor determination in soybean seed by multiple criteria 1. Crop Sci..

[51] Kharb R.S., Lather B.S., Deswal D.P. (1994). Prediction of field emergence through heritability and genetic advance of vigour parameters. Seed Sci. Technol..

[52] Thimmaiah S.R. (2012). Standard Methods of Biochemical Analysis.

[53] Sadasivam S. (1996). Biochemical Methods.

[54] Fokar M., Henry T.N., Blum A. (1998). Heat tolerance in spring wheat I. Estimating cellular thermo-tolerance and its heritability. Euphytica.

[55] Heath R.L., Packer L. (1968). Photoperoxidation in isolated chloroplasts: I. Kinetics and stoichiometry of fatty acid peroxidation. Arch. Biochem. Biophys..

[56] Livak K.J., Schmittgen T.D. (2001). Analysis of relative gene expression data using real-time quantitative PCR and the 2(-Delta C(T)) method. Methods.

[57] Gupta V.K., Mittal S.B. (1981). Evaluation of chemical methods for estimating available zinc and response of green gram (*Phaseolusaureus* roxb) to applied zinc in non-calcareous soils. Plant Soil.

[58] Ma L.Q., Komar K.M., Tu C., Zhang W., Cai Y., Kenelly E.D. (2001). A Fern that hyperaccumulates arsenic. Nature.

[59] Baker A.J.M., Brooks R.R. (1989). Terrestrial higher plants which hyperaccumulate metal elements. A review of their distribution, ecology and phytochemistry. Biorecovery.

[60] Watson D.J. (1952). The physiological basis of variation in yield. Adv. Agron..

[61] The Food and Agriculture Organization of the United Nations (FAO) (2002). Crops and Drops: Making the Best Use of Water for Agriculture.

[62] Minasny B., Hong S.Y., Hartemink A.E., Kim Y.H., Kang S.S. (2016). Soil pH increase under paddy in South Korea between 2000 and 2012. Agric. Ecosyst. Environ..

[63] Abd-Alla M.H., Nafady N.A., Bashandy S.R., Hassan A.A. (2019). Mitigation of effect of salt stress on the nodulation, nitrogen fixation and growth of chickpea (*Cicer arietinum* L.) by triple microbial inoculation. Rhizosphere.

[64] Ramadoss D., Lakkineni V.K., Bose P., Ali S., Annapurna K. (2013). Mitigation of salt stress in wheat seedlings by halotolerant bacteria isolated from saline habitats. SpringerPlus.

[65] Qurashi A.W., Sabri A.N. (2013). Osmolyte accumulation in moderately halophilic bacteria improves salt tolerance of chickpea. Pak. J. Bot..

[66] Shrivastava U.P., Kumar A. (2013). Characterization and optimization of 1-aminocyclopropane-1-carboxylate deaminase (ACCD) activity in different rhizospheric PGPR along with *Microbacterium* sp. strain ECI-12A. Int. J. Appl. Sci. Biotechnol..

[67] Essghaier B., Dhieb C., Rebib H., Ayari S., Boudabous A.R., Sadfi-Zouaoui N. (2014). Antimicrobial behavior of intracellular proteins from two moderately halophilic bacteria: Strain J31 of *Terribacillus halophilus* and strain M3-23 of *Virgibacillus marismortui*. J. Plant Pathol. Microbiol..

[68] Marakana T., Sharma M., Sangani K. (2018). Isolation and characterization of halotolerant bacteria and it’s effects on wheat plant as PGPR. Pharma Innov. J..

[69] Ahkami A.H., White R.A., Handakumbura P.P., Jansson C. (2017). Rhizosphere engineering: Enhancing sustainable plant ecosystem productivity. Rhizosphere.

[70] Six J., Frey S.D., Thiet R.K., Batten K.M. (2006). Bacterial and fungal contributions to carbon sequestration in agro-ecosystems. Soil Sci. Soc. Am. J..

[71] Wang S., Wu H., Qiao J., Ma L., Liu J., Xia Y., Gao X. (2009). Molecular mechanism of plant growth promotion and induced systemic resistance to tobacco mosaic virus by *Bacillus* spp.. J. Microbiol. Biotechnol..

[72] Zhang H., Kim M.S., Krishnamachari V., Payton P., Sun Y., Grimson M., Farag M.A., Ryu C.M., Allen R., Melo I.S. (2007). Rhizobacterial volatile emissions regulate auxin homeostasis and cell expansion in *Arabidopsis*. Planta.

[73] Egamberdieva D. (2009). Alleviation of salt stress by plant growth regulators and IAA producing bacteria in wheat. Acta Physiol. Plant..

[74] Pietri J.A., Brookes P.C. (2008). Relationships between soil pH and microbial properties in a UK arable soil. Soil Biol. Biochem..

[75] Boiero L., Perrig D., Masciarelli O., Penna C., Cassan F., Luna V. (2007). Phytohormone production by three strains of *Bradyrhizobium japonicum* and possible physiological and technological implications. Appl. Microbiol. Biotechnol..

[76] Egamberdieva D., Davranov K., Wirth S., Hashem A., Abd-Allah E.F. (2017). Impact of soil salinity on the plant-growth–promoting and biological control abilities of root associated bacteria. Saudi J. Biol. Sci..

[77] Olsen R.A., Bakken L.R. (1987). Viability of soil bacteria: Optimization of plate-counting technique and comparison between total counts and plate counts within different size groups. Microb. Ecol..

[78] Singh D., Rajawat M.V.S., Kaushik R., Prasanna R., Saxena A.K. (2017). Beneficial role of endophytes in biofortification of Zn in wheat genotypes varying in nutrient use efficiency grown in soils sufficient and deficient in Zn. Plant Soil.

[79] Velmourougane K., Prasanna R., Singh S., Chawla G., Kumar A., Saxena A.K. (2017). Modulating rhizosphere colonization, plant growth, soil nutrient availability and plant defense enzyme activity through *Trichoderma viride*-*Azotobacter chroococcum* biofilm inoculation in chickpea. Plant Soil.

[80] Singh D., Geat N., Rajawat M.V.S., Prasanna R., Kar A., Singh A.M., Saxena A.K. (2018). Prospecting endophytes from different Fe or Zn accumulating wheat genotypes for their influence as inoculants on plant growth, yield, and micronutrient content. Ann. Microbiol..

[81] Singh D., Geat N., Rajawat M.V.S., Mahajan M.M., Prasanna R., Singh S., Kaushik R., Singh R.N., Kumar K., Saxena A.K. (2018). Deciphering the Mechanisms of Endophyte-Mediated Biofortification of Fe and Zn in Wheat. J. Plant Growth Regul..

[82] Lucas J.A., García-Cristobal J., Bonilla A., Ramos B., Gutierrez-Manero J. (2014). Beneficial rhizobacteria from rice rhizosphere confers high protection against biotic and abiotic stress inducing systemic resistance in rice seedlings. Plant Physiol. Biochem..

[83] Wang Y., Yang X., Zhang X., Dong L., Zhang J., Wei Y. (2014). Improved plant growth and Zn accumulation in grains of rice (*Oryza sativa* L.) by inoculation of endophytic microbes isolated from a Zn hyperaccumulator, *Sedum alfredii* H.. J. Agric. Food Chem..

[84] White P.J., Broadley M.R. (2011). Physiological limits to zinc biofortification of edible crops. Front. Plant Sci..

[85] Naz I., Ahmad H., Khokhar S.N., Khan K., Shah A.H. (2016). Impact of zinc solubilizing bacteria on zinc contents of wheat. Am. Euras. J. Agric. Environ. Sci..

[86] Yadav R.C., Sharma S.K., Ramesh A., Sharma K., Sharma P.K., Varma A. (2020). Contribution of Zinc-Solubilizing and-Mobilizing Microorganisms (ZSMM) to enhance zinc bioavailability for better soil, plant, and human health. Rhizosphere Microbes.

[87] Yadav R., Ror P., Rathore P., Kumar S., Ramakrishna W. (2020). *Bacillus subtilis* CP4, isolated from native soil in combination with arbuscular mycorrhizal fungi promotes biofortification, yield and metabolite production in wheat under field conditions. J. Appl. Microbiol..

[88] Parida A.K., Das A.B. (2005). Salt tolerance and salinity effects on plants: A review. Ecotox. Environ. Safe..

[89] Ilangumaran G., Smith D.L. (2017). Plant growth promoting rhizobacteria in amelioration of salinity stress: A systems biology perspective. Front. Plant Sci..

[90] Meloni D.A., Oliva M.A., Martinez C.A., Cambraia J. (2003). Photosynthesis and activity of superoxide dismutase, peroxidase and glutathione reductase in cotton under salt stress. Environ. Exp. Bot..

[91] Hasegawa P.M., Bressan R.A., Zhu J.K., Bohnert H.J. (2000). Plant cellular and molecular responses to high salinity. Annu. Rev. Plant Biol..

[92] Zhang H., Murzello C., Sun Y., Kim M.S., Xie X., Jeter R.M., Zak J.C., Dowd S.E., Paré P.W. (2010). Choline and osmotic-stress tolerance induced in *Arabidopsis* by the soil microbe *Bacillus subtilis* (GB03). Mol. Plant Microbe Interact..

[93] Mahoney A.K., Yin C., Hulbert S.H. (2017). Community structure, species variation, and potential functions of rhizosphere-associated bacteria of different winter wheat (*Triticum aestivum*) cultivars. Front. Plant Sci..

[94] Gill S.S., Tuteja N. (2010). Reactive oxygen species and antioxidant machinery in abiotic stress tolerance in crop plants. Plant Phsiol. Biochem..

[95] Numan M., Bashir S., Khan Y. (2018). Plant growth promoting bacteriaas an alternative strategy for salt tolerance in plants: A review. Microbiol. Res..

[96] Johnson H.E., Broadhurst D., Goodacre R., Smith A.R. (2003). Metabolic fingerprinting of salt-stressed tomatoes. Phytochemistry.

[97] Hichem H., El Naceur A., Mounir D. (2009). Effects of salt stress on photosynthesis, PSII photochemistry and thermal energy dissipation in leaves of two corn (*Zea mays* L.) varieties. Photosynthetica.

[98] Meena K.K., Sorty A.M., Bitla U.M., Choudhary K., Gupta P., Pareek A., Singh D.P., Prabha R., Sahu P.K., Gupta V.K. (2017). Abiotic stress responses and microbe-mediated mitigation in plants: The omics strategies. Front. Plant Sci..

[99] Sharma P., Jha A.B., Dubey R.S., Pessarakli M. (2012). Reactive oxygen species, oxidative damage, and antioxidative defense mechanism in plants under stressful conditions. J. Bot..

[100] Prasad M., Srinivasan R., Chaudhary M., Jat L.K., Mukesh C., Lokesh Kumar J. (2019). Plant growth promoting rhizobacteria (PGPR) for sustainable agriculture: Perspectives and Challenges. PGPR Amelioration in Sustainable Agriculture.

[101] Singh S.B., Gowtham H.G., Niranjana S.R. (2019). ACC deaminase producing PGPR invoke changes in antioxidant systems to minimize the adverse effects of salt in sunflower. RJLBPCS.

[102] Bharti N., Pandey S.S., Barnawal D., Patel V.K., Kalra A. (2016). Plant growth promoting rhizobacteria *Dietzia natronolimnaea* modulates the expression of stress responsive genes providing protection of wheat from salinity stress. Sci. Rep..

[103] Larcher W. (1980). Physiological Plant Ecology: Ecophysiology and Stress Physiology of Functional Groups.

[104] Tester M., Davenport R. (2003). Na+ tolerance and Na+ transport in higher plants. Ann. Bot..

[105] Tiwari S., Singh P., Tiwari R., Meena K.K., Yandigeri M., Singh D.P., Arora D.K. (2011). Salt-tolerant rhizobacteria-mediated induced tolerance in wheat (*Triticum aestivum*) and chemical diversity in rhizosphere enhance plant growth. Biol. Fertil. Soils..

[106] Xu Z.H., Saffigna P.G., Farquhar G.D., Simpson J.A., Haines R.J., Walker S., Osborne D.O., Guinto D. (2000). Carbon isotope discrimination and oxygen isotope composition in clones of the F1 hybrid between slash pine and Caribbean pine in relation to tree growth, water-use efficiency and foliar nutrient concentration. Tree Physiol..

[107] Dodd I.C., Pérez-Alfocea F. (2012). Microbial amelioration of crop salinity stress. J. Exp. Bot..

[108] White P.J., Broadley M.R. (2001). Chloride in soils and its uptake and movement within the plant: A review. Ann. Bot..

[109] Alam S.M., Naqvi S.S.M., Ansari R.A. (1999). Impact of soil pH on nutrient uptake by crop plants. Handbook of Plant and Crop Stress.

[110] Al-Maliki S., AL-Masoudi M. (2018). Interactions between Mycorrhizal fungi, tea wastes, and algal biomass affecting the microbial community, soil structure, and alleviating of salinity stress in corn yield (*Zea mays* L.). Plants.

[111] Kumari S., Nee Sabharwal V.P., Kushwaha H.R., Sopory S.K., Singla-Pareek S.L., Pareek A. (2009). Transcriptome map for seedling stage specific salinity stress response indicates a specific set of genes as candidate for saline tolerance in *Oryza sativa* L.. Funct. Integr. Genomics..

[112] Deinlein U., Stephan A.B., Horie T., Luo W., Xu G., Schroeder J.I. (2014). Plant salt-tolerance mechanisms. Trends Plant Sc..

[113] Shahbaz M., Ashraf M. (2013). Improving salinity tolerance in cereals. Crit. Rev. Plant Sci..

[114] Zhang M., Cao Y., Wang Z., Wang Z.Q., Shi J., Liang X., Song W., Chen Q., Lai J., Jiang C. (2017). A retrotransposon in an *HKT1* family sodium transporter causes variation of leaf Na^+^ exclusion and salt tolerance in maize. New Phytol..

[115] Zhang M., Liang X., Wang L., Cao Y., Song W., Shi J., Lai J., Jiang C. (2019). A HAK family Na^+^ transporter confers natural variation of salt tolerance in maize. Nat.Plants.

[116] Jamil A., Riaz S., Ashraf M., Foolad M.R. (2011). Gene expression profiling of plants under salt stress. Crit. Rev. Plant Sci..

[117] Bouis H.E., Welch R.M. (2010). Biofortification—A sustainable agricultural strategy for reducing micronutrient malnutrition in the global South. Crop Sci..

[118] Zhang Y.Q., Sun Y.X., Ye Y.L., RezaulKarim M., Xue Y.F., Yan P., Meng Q.F., Cui Z.L., Cakmak I., Zhang F.S. (2012). Zinc biofortification of wheat through fertilizer applications in different locations of china. Field Crop Res..

[119] Cakmak I., Pfeiffer W.H., McClafferty B. (2010). Biofortification of durum wheat with zinc and iron. Cereal Chem..

[120] Cakmak I., McLaughlin M.J., White P. (2017). Zinc for better crop production and human health. Plant Soil.

[121] Francisco G.C., Poblaciones M.J., Almeida A.S., Cakmak I. (2016). Zinc (Zn) concentration of bread wheat grown under Mediterranean conditions as affected by genotype and soil/foliar Zn application. Plant Soil.

