# Supplementary material for: Salt-Tolerant Compatible Microbial Inoculants Modulate Physio-Biochemical Responses Enhance Plant Growth, Zn Biofortification and Yield of Wheat Grown in Saline-Sodic Soil
Source: Int J Environ Res Public Health. 2021 Sep 21;18(18):9936. doi: 10.3390/ijerph18189936 (PMC8467820; doi:10.3390/ijerph18189936)
Supplement: Supplementary file 1 [file ijerph-18-09936-s001.zip › ijerph-1358299-supplementary.pdf]

Supplementary Materials

Supplementary Table S1. Physico-biochemical properties of initial soil used in this study

S. No.	Soil parameters	Value
1.	Texture class	Dystric Eutrudepts (Inceptisols)
2.	Soil separates	
i.	Coarse sand (0.2–2.0 mm)	11.43%
ii.	Fine sand (0.02–0.2 mm)	17.50%
iii.	Silt (0.002–0.02 mm)	47.33%
iv.	Clay (<0.002 mm)	22.57%
3.	pH	8.2
4.	EC	1.8 dsM^{-1}
5.	ESP	7.67
6.	organic carbon	0.37%
7.	Olsen P	22.26 mg kg^{-1}
8.	total N	55.50 mg kg^{-1}
9.	K	126.50 mg kg^{-1}
10.	Zn	0.20 mg kg^{-1}

Supplementary Table S2. Salt tolerance (NaCl) and biochemical properties of selected strains used in this study

Properties/ characteristics	*B. amyloliquefaciens* B-16	*T. harzianum* UBST H-501
Amylase production	+ve	+ve
Salt tolerance (NaCl)	5.00%	4.50%
HCN production	+ve	+ve
IAA production	+ve	+ve
H_2O_2 production	+ve	ND
Urease test	+ve	ND
Catalase test	+ve	ND
Starch hydrolysis	+ve	+ve
Ammonia production	+ve	ND
Protease production	+ve	+ve
Siderophore production	+ve	+ve
Phosphate solubilization	+ve	+ve
Potash (K) solubilization	+ve	+ve
Zinc oxide (ZnO)	+ve (5.65 μg ml^{-1})*	+ve (4.50 μg ml^{-1})*
Zinc carbonate ($ZnCO_3$)	+ve (7.05 μg ml^{-1})*	+ve (5.69 μg ml^{-1})*
Zinc phosphate [$Zn_3(PO_4)_2$]	+ve (7.50 μg ml^{-1})*	+ve (6.90 μg ml^{-1})*

* Represents Zinc concentration (μg ml) in growth medium. Data are mean (n=5).
